# Differences in susceptibility of deciduous and permanent teeth to erosion exist, albeit depending on protocol design and method of assessment

**DOI:** 10.1038/s41598-022-08116-0

**Published:** 2022-03-09

**Authors:** Thiago Saads Carvalho, Adrian Lussi, Nadine Schlueter, Tommy Baumann

**Affiliations:** 1 Department of Restorative, Preventive and Pediatric Dentistry, School of Dental Medicine, University of Bern, Freiburgstrasse 7, 3010 Bern, Switzerland; 2 Department of Operative Dentistry and Periodontology, Center for Dental Medicine, Medical Center - University of Freiburg, Faculty of Medicine, University of Freiburg, Freiburg, Germany; 3 Division for Cariology, Department of Operative Dentistry and Periodontology, Center for Dental Medicine, Medical Center - University of Freiburg, Faculty of Medicine, University of Freiburg, Freiburg, Germany

**Keywords:** Preventive dentistry, Tooth erosion, Paediatric dentistry

## Abstract

Controversial results showing that deciduous teeth are more susceptible to erosion than permanent teeth might be related to study designs. We investigated how different conditions (pH: 3.0, 4.0, 5.0; acid agitation: gentle or vigorous; acid exposure times: 1–5 min) affect the susceptibility of both teeth to erosion. Enamel specimens (90 deciduous, 90 permanent) were distributed into groups (n = 15 permanent, n = 15 deciduous) according to acid pH (pH 5, 4 or 3) and agitation (gentle or vigorous) during erosive challenge. Both milder (less incubation time, gentle agitation, and higher pH) and more severe (longer incubation times, vigorous shaking, and lower pH) conditions were used. Demineralization was measured by relative surface microhardness (rSMH) and calcium released to the acid. Demineralization increased gradually for both teeth with increasing incubation time, agitation (gentle or vigorous), and with decreasing acid pH. The differences between deciduous and permanent teeth depended on the protocol design and assessment method. Under milder conditions, demineralization was better detectable with rSMH. Under more severe conditions, differences were more perceptible with calcium analyses. Differences exist in the susceptibility to erosion between deciduous and permanent teeth, but they are only distinguishable when the appropriate assessment method is used for the specific erosive condition.

## Introduction

Several factors can influence the rate of tooth demineralization during dental erosion, including those related to nutrition; such as the type, pH and concentration of mineral ions in the acid; or those related to the patient, like reflux, salivary factors, brushing, or behaviour, among many others^1,2^. Therefore, a vast number of in vitro and in situ studies have investigated the interplay between these factors, and a whole array of protocol designs have been proposed^3^. The variations in the protocol designs could therefore also be considered as a factor influencing the rate of tooth demineralization. This was demonstrated by Schlueter et al.^4^, who showed that one protocol design cannot be directly compared to another, and that the different models will lead to different outcomes. This can in turn lead to discrepancies between the studies.

Another parameter to be considered is the type of substrate. In case of using human teeth, it is often stated that there is a difference in the susceptibility of deciduous and permanent teeth to erosive demineralization. Deciduous and permanent enamel are morphologically and histologically different^5–8^, which would suggest that deciduous teeth have greater susceptibility to demineralization. This can be true regarding caries^7^, but the studies investigating erosive demineralization have shown conflicting results. Some studies show that deciduous teeth are more susceptible than permanent teeth^9–13^, but other studies see no differences^12,14–19^. These discrepancies might be due to different reasons, and we once proposed that specific standardized circumstances might be necessary for the differences to be detectable^20^. In fact, since protocol design influences tooth demineralization^4^, it is highly possible that the discrepancies are due to the different designs and demineralization procedures.

In this sense, the present study used different methods of assessment (surface microhardness or calcium analysis), different pH (3, 4, or 5), agitations (gentle or vigorous), and exposure times to the acid (range between 1 and 5 min), to create different erosive conditions (from mild to more severe conditions). The aim of this study was to investigate how these different conditions (protocol designs) affect the demineralization of deciduous and permanent teeth, observing if there are differences in their susceptibility to dental erosion.

## Materials and methods

### Preparation of the enamel specimens

Specimens were prepared as previously described^10^. From a pool of extracted human teeth that had been stored in chloramine solution, 180 molars (90 deciduous and 90 permanent) were cut using an Isomet® low speed saw (Isomet, Buehler Ltd., Düsseldorf, Germany) to obtain enamel specimens. These were embedded in acrylic resin (Paladur®; Heraeus Kulzer GmbH, Hanau, Germany) and then ground using a Knuth Rotor machine (Labpol 21, Struers, Copenhagen, Denmark) with silicon carbide paper discs of grain size 18.3 µm, 8 µm, 5 µm for 60 s each, removing a total of 200 µm of the top surface layer of the enamel. Between these steps, the specimens were rinsed and sonicated for 1 min in water. Further polishing with 3 µm diamond abrasive was carried out for 60 s (LaboPol-6, DP-Mol Polishing, DP-Stick HQ, Struers). The flat and polished enamel specimens were stored in a mineral solution (1.5 mmol/l CaCl_2_, 1.0 mmol/l KH_2_PO4 50 mmol/l NaCl, pH = 7.0) at 4 °C until the time of the experiment. This was performed in order to keep the teeth in a medium with mineral equilibrium, and no demineralization would occur until the start of the experiment. This, on the other hand, could cause some deposition of minerals onto the enamel surface. To eliminate this effect, we performed a final polishing with 1 µm diamond abrasive paste under constant cooling (LaboPol-6, DP-Mol Polishing, DP-Stick HQ, Struers) and then sonicated for 1 min prior to the start of the experiment.

The 90 specimens from permanent and the 90 specimens from deciduous enamel were randomly distributed into five groups per substrate (n = 15 permanent and n = 15 deciduous) according to the acid pH (pH of 5, 4 or 3) and the agitation during erosive challenge (gentle or vigorous).

### Experimental procedure

Initially, the baseline surface microhardness (SMH) of all specimens was measured. Later, the specimens were submitted to an erosive challenge with citric acid. The citric acid was prepared by dissolving citric acid monohydrate in deionized water (10.937 g/l; 1% concentration), and the pH of the acid was adjusted with KOH 1 M, according to the groups to pH 5, pH 4 or pH 3. The erosive challenge was made in water baths with reciprocating movement set at 70 oscillations/min and at a constant temperature (25 °C), but the shaking movement was different with respect to the travel paths of 22 mm and 50 mm. These different travel paths caused to different shaking speeds of ≈ 52 mm/s (gentle shaking) and ≈ 116 mm/s (vigorous shaking). Each erosive challenge was made by incubating the enamel specimens for 1 min in 10 mL citric acid, and new aliquots of acid solutions were taken for every challenge.

After the erosive challenge, the specimens were washed, dried with air, and taken for SMH measurements, while the citric acid was stored for later calcium analyses. The erosive challenge and measurements were repeated 5 times.

### Surface microhardness (SMH) measurements

Specimens were prepared as previously described^10^. Knoop SMH measurements were performed with a load of 50 g and a dwell time of 10 s (UHL VMHT Microhardness Tester, UHL technische Mikroskopie GmbH & Co. KG, Aßlar, Germany). After each erosive challenge, six impressions were made with 25 µm distance between them, and their length was measured. The hardness value of each impression was calculated by an integrated software of the device, and the average from the six impressions was taken as the SMH value and was considered for analysis. The SMH was transformed to relative SMH (rSMH), considering the baseline value as 100%, and rSMH was used for statistical analysis and interpretation. The rSMH was calculated using the formula: rSMH = (SMH_i_/SMH_baseline_) × 100, where SMH_baseline_ is the initial microhardness value and SMH_i_ is the hardness value of the ith erosive challenge (i = 1, 2, 3, 4 or 5).

### Calcium released to the citric acid

To analyze calcium released to the citric acid, we used an atomic absorption spectrometer with acetylene-air flame (AAnalyst 400, Perkin Elmer Analytical Instruments, EUA). Standard calcium concentrations were used to obtain a standard curve and calibrate the equipment. To eliminate the interference of phosphate ions, lanthanum nitrate was added to the citric acid and to the standards (final concentration of 0.5% w/v). Once the amount of calcium was measured in the citric acid, the values were normalized to the surface area of the enamel specimens. For that, the enamel surface area was measured with a light microscope (Leica, M420) connected to a fixed camera (Leica, DFC495), and automatically calculated with the software program IM500. For statistical analyses, the results are presented in nmol of Calcium/mm^2^ of enamel.

### Ethical statement

The teeth used in this study were from a pooled biobank, so the local ethic committee (KEK—Kantonale Ethikkommission Bern, Bern, Switzerland) categorizes them as irreversibly anonymized, and no previous ethical approval was necessary. In accordance with the guidelines and regulations of the KEK, the volunteers were informed about the use of their teeth in research and their oral consent was obtained.

### Statistics

Normal distribution was verified with the Shapiro–Wilk’s test, and the data was not normally distributed. Initially, to verify the interactions between the parameters, we performed Generalized Linear Models (GdLMs). Since the data was not normally distributed, a comprehensive analyses including time-related factor (erosion time) was not possible, so separate GdLMs were made for each erosion time (1–5 min). Both rSMH and calcium measurements were used as dependent variables, and the parameters were considered as fixed factors: pH (pH 3, pH 4 and pH5), agitation (gentle and vigorous shaking), and tooth types (deciduous and permanent). Afterwards, Kruskal–Wallis tests with post-hoc Dunn’s Tests, as well as Mann–Whitney tests were performed, considering the final rSMH and final calcium analyses (after 5 min incubation in acid). Bonferroni corrections were performed for multiple testing.

## Results

**Figure 1:**
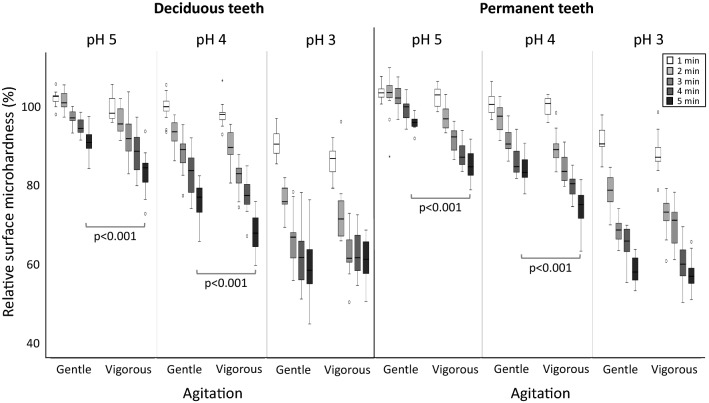
Box-plots for the relative surface microhardness (rSMH, %) of deciduous and permanent teeth, according to the incubation time in citric acid (1 min to 5 min, different shaded boxes), the pH of the citric acid (pH 5, pH 4 or pH 3), and the agitation (gentle or vigorous shaking). Significant differences between gentle and vigorous shaking after 5 min incubation are shown when *p* < 0.05.

**Figure 2:**
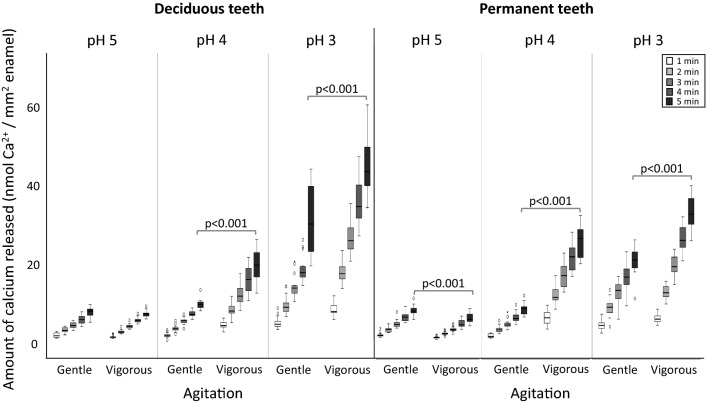
Box-plots for the amount of calcium (nmol of Ca^2+^/mm^2^ of enamel) released from deciduous and permanent teeth, according to the incubation time in citric acid (1 min to 5 min, different shaded boxes), the pH of the citric acid (pH 5, pH 4 or pH 3), and the agitation (gentle or vigorous shaking). Significant differences between gentle and vigorous shaking after 5 min incubation are shown when *p* < 0.05.

Figures 1 and 2 summarize the relative surface microhardness and the calcium release results, respectively, with the box-plots representing each erosive challenge (1 to 5 min), for each shaking movement, each acid pH and tooth type. Both figures show the results for deciduous (left) and permanent teeth (right). Examining each half of the figures (each type of tooth) from left to right, we observe a gradual intensification of the enamel demineralization. This occurred with the increase in incubation times (from 1 to 5 min), with the increase in shaking strength (from gentle to vigorous), and with the decrease in pH of the acid (from pH 5 to pH 3). These conditions caused the teeth to progressively demineralize, leading to greater decreases in surface hardness (Fig. 1) and more calcium being released from the enamel (Fig. 2).

The *p* values from the GdLMs are presented in Tables 1 and 2, showing the interaction of the factors with the outcome variables. Table 1 shows that rSMH was significantly affected by the different factors individually, except factor “tooth” at rSMH_3min_, but when analyzed together (in interaction), some factors affected rSMH more than others. The interaction of tooth type with either pH (Tooth*pH) or agitation (Tooth*Agitation) caused lesser effect at initial erosion times, and more significant effects at rSMH_5min_. Both pH and Agitation had greater effects on rSMH, but when tooth was added to the model (Tooth*pH*Agitation) a more significant effect was observed only at rSMH_3min_.

**Table 1:** Significance values for the impact of individual factors, as well as their interaction, on the relative surface microhardness (rSMH) at each erosion time (1–5 min).

Factor	rSMH_1min_	rSMH_2min_	rSMH_3min_	rSMH_4min_	rSMH_5min_
**Individual**					
Tooth	< **0.001**	0.071	< **0.001**	**0.019**	< **0.001**
pH	< **0.001**	< **0.001**	< **0.001**	< **0.001**	< **0.001**
Agitation	< **0.001**	< **0.001**	< **0.001**	< **0.001**	< **0.001**
**Interactions**					
Tooth*pH	0.785	0.585	0.261	0.344	< **0.001**
Tooth*agitation	0.176	**0.031**	0.468	**0.004**	**0.041**
pH*agitation	**0.032**	0.896	< **0.001**	< **0.001**	< **0.001**
Tooth*pH*agitation	0.980	0.405	**0.002**	0.157	0.972

Significant values are in [bold].

**Table 2:** Significance values for the impact of individual factors, as well as their interaction, on the calcium release (CaR) at each erosion time (1–5 min).

Factor	CaR_1min_	CaR_2min_	CaR_3min_	CaR_4min_	CaR_5min_
**Individual**					
Tooth	0.372	0.187	0.079	**0.019**	< **0.001**
pH	< **0.001**	< **0.001**	< **0.001**	< **0.001**	< **0.001**
Agitation	< **0.001**	< **0.001**	< **0.001**	< **0.001**	< **0.001**
**Interactions**					
Tooth*pH	< **0.001**	< **0.001**	< **0.001**	< **0.001**	< **0.001**
Tooth*agitation	0.898	0.568	0.600	0.373	0.290
pH*agitation	< **0.001**	< **0.001**	< **0.001**	< **0.001**	< **0.001**
Tooth*pH*agitation	< **0.001**	< **0.001**	< **0.001**	< **0.001**	**0.006**

Significant values are in [bold].

Table 2 shows how calcium release was significantly affected by the different factors. Both pH and agitation individually affected calcium release, but tooth type had less of an effect at initial erosion times (CaR_1min_–CaR_3min_) with greater effect from 4 min erosion onward. The interaction of tooth and agitation had less effect, but when pH was included in the model (pH*Tooth; pH*Agitation, and pH*Tooth*Agitation) a significant effect was observed.

The results from the Kruskal–Wallis tests are observed in Figs. 1 and 2, showing the differences between the factor groups at rSMH_5min_ and CaR_5min_. A clear effect of pH can be observed (*p* < 0.001), with more demineralization occurring at lower pH. The effect of shaking depended on the type of tooth and on the pH of the citric acid (Figs. 1, 2), corroborating the Tooth*Agitation and pH*Agitation interactions for rSMH, and pH*Agitation for CaR.

Looking specifically at deciduous teeth, the citric acid with pH 4 caused significantly more demineralization when vigorous shaking was used than with gentle shaking (*p* < 0.001). In milder acid challenges (at pH 5), differences between gentle and vigorous shaking were observed in rSMH results (*p* < 0.001), but not in calcium release (*p* = 0.912); whereas in more severe challenges (at pH 3), differences were observed in calcium release (*p* < 0.001), but not in the rSMH results (*p* = 1.000). Likewise for permanent teeth, where the differences between gentle and vigorous shaking were more distinctly observed in rSMH, when the acid challenge was milder (pH 4 or pH 5, *p* < 0.001); whereas the differences were more clearly observed in the calcium results, when the acid challenge was more severe (pH 4 and pH 3; *p* < 0.001).

**Figure 3:**
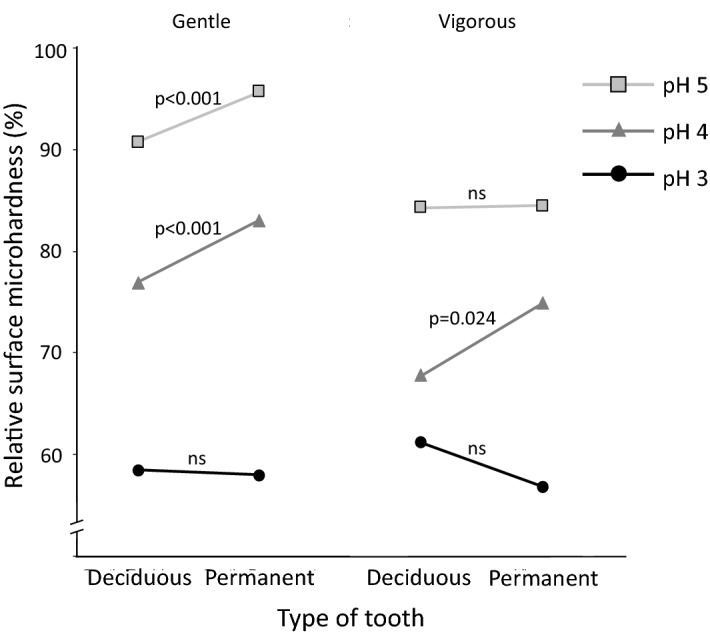
Differences in the relative surface microhardness (rSMH, %) between deciduous and permanent teeth, according to different pH values [pH 5 (squares), pH 4 (triangles) or pH 3 (circles)] and different agitations [gentle (left) or vigorous (right) shaking]. Significant differences between deciduous and permanent teeth are shown when *p* < 0.05, and “ns” is given when difference is not significant. To better visualize the results, the deviation values are not given here, but the complete data is displayed in Fig. 1.

**Figure 4:**
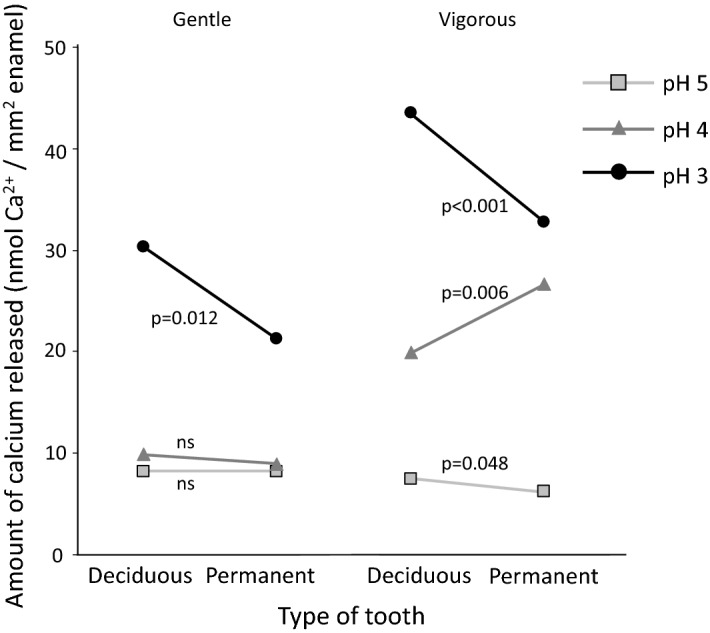
Differences in the amount of calcium (nmol of Ca^2+^/mm^2^ of enamel) released from deciduous and permanent teeth, according to different pH values [pH 5 (squares), pH 4 (triangles) or pH 3 (circles)] and different agitations [gentle (left) or vigorous (right) shaking]. Significant differences between deciduous and permanent teeth are shown when *p* < 0.05, and “ns” is given when difference is not significant. To better visualize the results, the deviation values are not given within this figure, but the complete data is displayed in Fig. 2.

For better visualization, the differences between deciduous and permanent teeth are illustrated in Figs. 3 and 4. At the end of the experiment, deciduous teeth seem to have demineralized more than permanent teeth, where they generally had significantly lower rSMH than permanent teeth, and generally released more calcium. However, the detection of this difference between the two kinds of teeth depended on the severity of the acid challenge and on the method of assessment (rSMH or calcium release). rSMH could better detect the differences when the erosive challenges were milder, i.e. with gentle shaking and citric acid at pH 5 or pH 4 (*p* < 0.001; Fig. 3); whereas the calcium results could better show this difference when the challenge was more severe, i.e. with citric acid at pH 3, either with gentle or vigorous shaking (*p* < 0.05; Fig. 4).

## Discussion

This study tested deciduous and permanent teeth considering different protocol designs and methods of assessment, to establish an array of demineralization conditions, ranging from milder erosive conditions (shorter exposure times to acid, higher pH, and gentle shaking) to more severe conditions (longer exposure times, lower pH, and vigorous shaking). We detected significant differences between deciduous and permanent teeth, but previous studies have reported conflicting results^21,22^. These discrepancies have been attributed to the different sample sizes used in the experiments, or the different conditions^12,20^. In clinical studies, however, the rate of demineralization seem to be faster in deciduous teeth than in permanent teeth^21^. Although in clinical situations abrasion (from tooth brushing) and attrition (from grinding teeth) also play relevant roles in erosive tooth wear, the present study now partly clarifies the divergence in susceptibility between deciduous and permanent teeth.

The most obvious differences between deciduous and permanent teeth are in their anatomy, where deciduous teeth are smaller and have a thinner and more porous enamel layer^6^. Although both kinds of teeth have similar enamel prisms^23,24^, the prisms in deciduous teeth are smaller, more curved, and more widely spread^7,8^. Moreover, their hydroxyapatite crystals are considerably different. The crystals found in both permanent and deciduous enamel are imperfect forms of hydroxyapatite, basically made up of calcium (Ca^2+^), phosphate (PO_4_^3−^) and hydroxyl (OH^−^) ions with some ‘impurity’ ions, such as fluoride (F^−^), carbonate (CO_3_^2−^) and sodium (Na^+^)^25^, in a crystalline structure with the simplified chemical formula: Ca_10–x_Na_x_(PO_4_)_6–y_(CO_3_)_z_(OH)_2–u_F_u_^25^. An important impurity ion in the difference between deciduous and permanent enamel is carbonate (CO_3_^2−^). When CO_3_^2−^ is present in the apatite crystal, this carbonated hydroxyapatite causes a distorted lattice conformation and the crystals are more soluble than the stoichiometric hydroxyapatite. Since deciduous enamel contains greater total CO_3_^2−^ content^26^, it is therefore more susceptible to dissolution. Additionally, deciduous enamel is less mineralized than its permanent counterpart^5^, it presents greater enamel porosity, greater organic content in the enamel structure^27^, and, consequently, lower elasticity and lower surface microhardness^15^. Therefore, chemically and histologically, deciduous teeth have all the attributes for a greater rate of demineralization than permanent teeth.

Indeed, our results generally corroborated this fact. We further observed that, at milder conditions, these differences were more noticeable with the surface microhardness measurements, whereas in more severe conditions, the differences were observed in the calcium results. This reflects the histopathology of erosive demineralization. At milder conditions, the demineralization occurs at a slower rate and the partial loss of mineral causes an initial softening to the enamel surface. Consequently, rSMH detected the differences between the two kinds of teeth at milder conditions, but the amount of calcium released was still too low, and the variations were high enough to hinder the detection of any significant differences between both kinds of teeth. As the severity of the conditions intensified, the softened layer on both deciduous and permanent enamel reached a saturation point^28^, with no further change to their surface hardness. This can be observed in Fig. 1, especially at pH 3, where the rSMH tends to level off at later erosion times (especially with deciduous teeth), and there is no difference between gentle and vigorous shaking. Therefore, rSMH can no longer detect the differences at more severe conditions, but calcium continues to be released to the acid, and the differences between the two kinds of teeth can be detected with this method of assessment. Despite the greater calcium release from permanent teeth at pH 4 vigorous shaking (to which we currently have no explanation), our results suggest that the lack of differences between the two kinds of teeth observed in the above-mentioned studies^14–19^ could actually be due to the method of assessment adopted with respect to the conditions used. This means that some methods can better detect the differences at milder demineralization conditions, while other methods are better for more severe conditions^29,30^.

The different conditions used in our study were reached by varying the pH, one of the most important chemical factors in dental erosion^25^, and the agitation, one of the most important physical factors^31^. Both these factors played major roles in enamel dissolution in our study; furthermore, the interaction of both is of particular relevance as shown by the regression analysis. During enamel dissolution, the H^+^ ions (determined by the pH) will drive the outflow of Ca^2+^, PO_4_^3−^, OH^−^ and CO_3_^2−^ ions from the enamel to the acid. If there is no agitation, this acid remains almost stagnant in the system, and the layer of acid in the immediate vicinity of the enamel (the Nernst layer) remains semi-static. This allows for the slow neutralization of the H^+^ ions within the Nernst layer by the PO_4_^3−^, OH^−^ and CO_3_^2−^ released from the demineralized enamel^32^. This slight rise in the local pH will reduce the demineralization rate. However, during more vigorous agitation, the Nernst layer will be dispersed and renewed, leading to a constant exchange of Ca^2+^, PO_4_^3−^, OH^−^ and CO_3_^2−^ ions with the medium, and new H^+^ ions from the acid will be available to drive the process forward, generating more demineralization. This process is intensified, if the pH of the solution is lowered, leading to an absolute increase in H^+^ ions.

Drawing a parallel to clinical situations, the more severe conditions used in the present study could represent patients who swish drinks in their mouths (vigorous agitation) or who hold drinks in their mouths (longer time of acid exposure)^31^ consuming either drinks with higher pH such as for e.g. orange juice or with lower pH such as soft drinks or energy drinks. A clinical study has already observed that patients generating these severe conditions with their drinking behavior also presented more erosive tooth wear^33^. In the present study, however, we did not include other important variables, such as the type of acid in the acidic substance^34,35^, presence of calcium and phosphate ions in the substance^34,36,37^, or viscosity of the substance^20,38,39^. It would be virtually impossible to design a study controlling and comparing all these variables at once.

Other important clinical factors affecting erosive tooth wear are saliva^10,40,41^ and toothbrushing^13,42^. Saliva of children contains significant lower calcium^43^ and protein concentrations^44^, with an apparent linear increase in total protein concentrations with age^45^. This is probably related to the thinner salivary pellicle formed on deciduous teeth^41^, which contains less than half of the proteins in common with the pellicle formed on permanent teeth^40^. Despite these differences, adult saliva better protects permanent teeth, whereas saliva from children better protects deciduous teeth^10^. Regarding tooth wear, toothbrushing is a substantial factor that could accelerate the wear of both kinds of teeth. If deciduous teeth are more susceptible to erosive demineralization, this could also mean that they present faster wear rate than its permanent pendant. This was observed in some studies, where erosive tooth wear in deciduous teeth was greater than in permanent teeth^13,46^, but another study observed no differences between both kinds of teeth^14^. Again, different study designs were used, which could account for the difference in the results.

Hunter and coworkers^11^ once suggested that the differences between deciduous and permanent teeth might rather appear over time; and we had similarly proposed that, given the specific circumstances, these differences would be more distinguishable^20^. Now, our results show that the differences exist, but are observable depending on the conditions used and the methods of assessment. If the conditions are milder, where only initial changes to the enamel surface occur, surface analyses, such as hardness or roughness, might be more suitable. However, as the severity intensifies, the softened layer will reach a steady state, and the differences will no longer be perceptible. Therefore, at more severe conditions, other methods will be more appropriate, such as calcium analyses or profilometry. If, however, the latter methods are used at milder conditions, or the former methods used in severe conditions, the noise in the results might mask the differences between the two groups. Furthermore, if other factors are introduced into the protocol design, such as saliva or toothbrushing, they will have different effects on the different enamel substrates, thereby affecting their demineralization rates distinctively. Hence, the conditions used will also play a role.

Given the innumerous variables influencing erosive demineralization, it is virtually impossible to make a direct comparison between the different studies, especially when the methodological details are not explicit in the publications. The different models and methodologies used in such studies have already been previously discussed^3^, but no specific guidelines were proposed for designing “the most appropriate experimental model”. In fact, any methodological condition will be highly dependent on the objectives of each individual study. What is most important is that authors are urged to spare no methodological details when reporting their methods. Although these minutiae might seem trivial (like acid pH, concentration, agitation methods, temperatures, durations, etc.), they will greatly influence the results and might even explain the divergences across the different studies. Moreover, the ideal method for measurement will greatly depend on the characteristics of the tooth surface, on the stage of the erosive lesion, as well as on the expected changes to the structure of the lesion^30^. So, in-depth understanding of the histopathological aspects of the demineralization process is the decisive factor when planning an experiment.

We conclude that differences in the susceptibility to erosion between deciduous and permanent teeth do exist, but they are only distinguishable provided that the methods of assessment are appropriate for the conditions used, taking into consideration the protocol design.

## Data Availability

We agree to make available all the materials, data and associated documents to readers.
